# Optical projection tomography reveals dynamics of HEV growth after immunization with protein plus CFA and features shared with HEVs in acute autoinflammatory lymphadenopathy

**DOI:** 10.3389/fimmu.2012.00282

**Published:** 2012-09-07

**Authors:** Varsha Kumar, Susan Chyou, Jens V. Stein, Theresa T. Lu

**Affiliations:** ^1^Autoimmunity and Inflammation Program and Pediatric Rheumatology, Hospital for Special SurgeryNew York, NY, USA; ^2^Theodor Kocher Institute, University of BernBern, Switzerland; ^3^Department of Microbiology and Immunology, Weill Medical College of Cornell UniversityNew York, NY, USA

**Keywords:** lymph node, high endothelial venules, stromal, angiogenesis, optical projection tomography, autoimmunity, autoinflammation, regulatory T cells

## Abstract

The vascular–stromal compartment of lymph nodes is important for lymph node function, and high endothelial venules (HEVs) play a critical role in controlling the entry of recirculating lymphocytes. In autoimmune and autoinflammatory diseases, lymph node swelling is often accompanied by apparent HEV expansion and, potentially, targeting HEV expansion could be used therapeutically to limit autoimmunity. In previous studies using mostly flow cytometry analysis, we defined three differentially regulated phases of lymph node vascular–stromal growth: initiation, expansion, and the re-establishment of vascular quiescence and stabilization. In this study, we use optical projection tomography to better understand the morphologic aspects of HEV growth upon immunization with ovalbumin/CFA (OVA/CFA). We find HEV elongation as well as modest arborization during the initiation phase, increased arborization during the expansion phase, and, finally, vessel narrowing during the re-establishment of vascular quiescence and stabilization. We also examine acutely enlarged autoinflammatory lymph nodes induced by regulatory T cell depletion and show that HEVs are expanded and morphologically similar to the expanded HEVs in OVA/CFA-stimulated lymph nodes. These results reinforce the idea of differentially regulated, distinct phases of vascular–stromal growth after immunization and suggest that insights gained from studying immunization-induced lymph node vascular growth may help to understand how the lymph node vascular–stromal compartment could be therapeutically targeted in autoimmune and autoinflammatory diseases.

## INTRODUCTION

Lymph nodes are sites of immune responses, and the lymph node blood vessels are an integral part of immune function. In addition to the delivery of oxygen and micronutrients, the lymph node blood vessels deliver lymphocytes and other immune cells to the lymph node parenchyma, where these cells can interact with antigen and one another to generate an adaptive immune response. Recirculating lymphocytes enter the lymph node parenchyma at specialized postcapillary venules characterized by cuboidal, rather than flat, endothelial cells that are known as high endothelial venules (HEVs). The HEV endothelial cells display chemokines and adhesion molecules that promote the extravasation of lymphocytes. Peripheral lymph node HEVs are identified in part by their expression of peripheral node addressin (PNAd) which binds L-selectin on recirculating lymphocytes and thus allowing lymphocyte entry (von Andrian and Mempel, 2003; Benahmed et al., 2012). The HEVs and the vessels in general are suspended within a conduit network consisting of collagen-rich fibrils ensheathed by fibroblastic reticular cells (FRCs). The conduit network is functionally connected to the blood vessels, with FRCs or related pericyte-like cells ensheathing the vessel walls (Gretz et al., 1997; Malhotra et al., 2012). Disrupting trafficking across HEVs disrupts lymphocyte accumulation in lymph nodes and delays immune responses (Arbones et al., 1994; Steeber et al., 1996; Xu et al., 1996). Thus, insight into the events and regulation of HEV alterations during immune responses may lead to new ways by which to therapeutically manipulate immunity.

Lymph node enlargement induced by immunization or infection is associated with vascular expansion (Burwell, 1962; Herman et al., 1972; Anderson et al., 1975; Mebius et al., 1990; Webster et al., 2006; Kumar et al., 2010). Studies in recent years have begun to delineate the mechanisms that regulate the growth of HEV and other portions of the vasculature (Soderberg et al., 2005; Angeli et al., 2006; Liao and Ruddle, 2006; Webster et al., 2006; Kumar et al., 2010; Kataru et al., 2011). From our studies of lymph node vascular growth after immunization, we have identified at least three phases based on the vascular characteristics and regulatory mechanisms. During the initiation phase from day 0 to day 2, there is rapid upregulation of endothelial cell proliferation which is independent of T and B cells, a modest increase in HEV endothelial cell numbers, but no expansion of non-HEV blood endothelial cells. The initiation phase appears to involve at least a tripartite interaction whereby dendritic or other CD11c^+^ cells stimulate FRCs to upregulate VEGF, which then promotes endothelial cell proliferation. The initiation phase is followed by an expansion phase from day 2 to about day 7 after ovalbumin/CFA (OVA/CFA), and it is characterized by continued upregulated proliferation, expansion of HEV, non-HEV blood, and lymphatic endothelial cell numbers, expansion of FRC numbers, and dependence of the proliferation and expansion on T and B cells (Webster et al., 2006; Tzeng et al., 2010; Chyou et al., 2011; Benahmed et al., 2012). After expansion, the vessels become more quiescent, with reduced endothelial cell proliferation and HEV trafficking efficiency. Stimulating the lymph node transiently with bone marrow-derived dendritic cells leads to cessation of vascular expansion at this stage, but a chronic stimulus such as with OVA/CFA leads to continued expansion as the proliferation rate slowly drops. Re-establishment of vessel stabilization accompanies the quiescence, with FRCs more tightly organized around HEVs. The quiescence and stabilization are mediated by CD11c^hi^ presumed dendritic cells, as depletion of CD11c^hi^ cells during this phase results in increased endothelial cell proliferation, disorganization of the FRC sheath around the vessels, and increased vascular permeability (Tzeng et al., 2010).

In autoimmune and autoinflammatory disorders such as systemic lupus erythematosus and systemic onset juvenile arthritis, lymph nodes can be hypertrophic (Fox and Rosahn, 1943; Shapira et al., 1996; Schneider and Laxer, 1998; Calguneri et al., 2003; Livingston et al., 2011). Apparent blood vascular proliferation is one of the characteristics of these enlarged lymph nodes (Benahmed et al., 2012). We recently showed in a chronic lupus model that the expanded lymph node vasculature is in a state of re-established quiescence as seen by a low endothelial cell proliferation rate, reduced HEV trafficking efficiency, and the accumulation of CD11c^hi^ cells (Chyou et al., 2012). Among other things, these results suggested that insights gained from studying lymph nodes stimulated by model immunization strategies may be applicable to vascular regulation in chronic autoimmune models and, potentially, in human disease. While the lymph nodes in the chronic lupus model steadily enlarged over weeks (Chyou et al., 2012), lymph nodes in human lupus and systemic onset juvenile arthritis can also acutely enlarge (Fox and Rosahn, 1943; Eisner et al., 1996; Grom and Passo, 1996). Whether acute lymphadenopathy associated with autoimmunity or autoinflammation can be accompanied by an expanded vasculature and whether this expansion shares features with acute vascular expansion induced by OVA/CFA immunization is unknown.

Blood vessel expansion in immune-stimulated lymph nodes was demonstrated decades ago using techniques such as resin casting and microangiography (Burwell, 1962; Herman et al., 1972; Anderson et al., 1975; Steeber et al., 1987). Optical projection tomography (OPT) is an imaging technique developed 10 years ago whereby the specimen is rotated while a fixed detector obtains light or fluorescent images through the course of the 360^°^ rotation, allowing a three-dimensional rendering of the specimen. The apparatus for OPT data capture displays many similarities to a micro-computer tomography (CT) scanner: a stepper motor is used to rotate the specimen to precisely controlled angles, a 2D array detector (CCD) is used to capture the signal, and photon sources are included for illumination. OPT generally employs relatively low numerical aperture optics, which provide the large depth of field and working distance needed to analyze thick samples. The use of visible light and image-forming optics is one of the important advantages of OPT – it is capable of being used in two different modalities: transmission OPT (tOPT), and emission OPT (eOPT). The use of optics in OPT allows a sharp image to be focused onto the CCD, yielding images that can be considered good approximations to projections for computational purposes. OPT is ideal for specimens 1–10 mm in size (Sharpe et al., 2002) and has been used in studies of organ development during embryogenesis (Davies and Armstrong, 2006). Recently, OPT was used to study HEV growth associated with virus-induced lymph node hypertrophy (Kumar et al., 2010).

In this current study, we use OPT to characterize the morphologic aspects of HEV growth upon immunization with OVA/CFA and compare that with the HEV alterations that occur in an acute autoimmune and autoinflammatory model induced by depletion of regulatory T cells. The results show (1) distinct morphologic alterations during the different phases of vascular growth after immunization, (2) that HEVs in acutely enlarged autoinflammatory lymph nodes are expanded, and (3) that there are morphologic similarities between expanded HEVs in the two models. These results reinforce the idea of distinct phases of lymph node vascular growth after immunization and raise the possibility that insights gained from studying lymph nodes stimulated by OVA/CFA and other experimental immunization strategies may be useful in understanding and treating autoimmune and autoinflammatory diseases.

## MATERIALS AND METHODS

### MICE

C57Bl/6 mice between 6 and 12 weeks of age were obtained from Jackson Laboratory (Bar Harbor, ME, USA), Taconic Farms (Hudson, NY, USA) or National Cancer Institute (Frederick, MD, USA). Foxp3-DTR mice were originally generated as described (Kim et al., 2007). All procedures were performed in accordance with the Institutional Animal Use and Care Committee of the Hospital for Special Surgery.

### MOUSE TREATMENTS AND IMMUNIZATIONS

For immunization with OVA in complete Freund’s adjuvant (OVA/CFA), OVA was emulsified in CFA at final concentration of 1 mg/ml OVA, and mice received hind footpad injection of 20 μl of OVA/CFA as described (Webster et al., 2006).

Foxp3-DTR mice express simian diphtheria toxin (DT) receptor that allows for depletion of Foxp3^+^ regulatory T cells upon DT administration (Kim et al., 2007; Chinen et al., 2010). For regulatory T cell depletion, wild-type control or Foxp3-DTR mice were injected intraperitoneally with 1.5 μg of DT (Calbiochem, San Diego, CA, USA or List Biological Laboratory, Campbell, CA, USA) on days 0, 1, 4, and 7 and examined on day 8. CD4^+^, CD25^+^, Foxp3^+^ regulatory T cells in lymph nodes were depleted by at least 12-fold by day 8.

### FLOW CYTOMETRY ANALYSIS

For flow cytometric analysis of endothelial cells and FRCs, we generated single cell suspensions from lymph nodes and stained cells as previously described (Webster et al., 2006). Briefly, lymph nodes were digested with collagenase type II (Worthington, Lakewood, NJ, USA) and stained with antibodies for flow cytometry. Antibodies to the following antigens were used: CD45 (BD Biosciences, San Jose, CA, USA), CD31 (BD Biosciences), PNAd (clone MECA-79, BD Biosciences), gp38 (Biolegend, San Diego, CA, USA or Developmental Studies Hybridoma Bank, Iowa City, IA, USA). The unconjugated anti-PNAd is detected using fluorophore-conjugated anti-rat IgM (Jackson ImmunoResearch, West Grove, PA, USA). Cells were analyzed using a FACS CANTOS (BD Biosciences) and CellQuest Pro (BD Biosciences) software.

### OPTICAL PROJECTION TOMOGRAPHY

MECA-79 conjugation was performed according to vendor instructions using Alexa-568 conjugation kit (Invitrogen, Carlsbad, CA, USA) and visualization of the HEVs by OPT (eOPT modality) was performed essentially as described in Kumar et al. (2010). To label the HEVs, 15 μg of Alexa-568-conjugated MECA-79 was injected intravenously into the mouse at 20 min prior to lymph node excision. Excised lymph nodes were cleaned of fat, fixed in 1% ultrapure paraformaldehyde (Electron Microscopy Sciences, Hatfield, PA, USA) for 4 h at 4^°^C and then washed in PBS. The lymph nodes were embedded in 1% ultrapure agarose (Invitrogen) and then dehydrated in 100% methanol overnight. The lymph nodes were then immersed in BABB solution (benzyl alcohol + benzyl benzoate at 1:2 ratio) and OPT scanning was performed according to the manufacturer’s instructions (Bioptonics). Filter sets were: exciter 425/40, emitter LP475 for autofluorescent signal and exciter 545/30, emitter 617/75 for red fluorescent signal. Raw data were converted into 3D voxel datasets using NRecon software from Bioptonics. Reconstructed virtual xyz data sets were exported as .TIFF or .bmp files and analyzed with IMARIS (Bitplane) for isosurface calculation of total lymph node volume. IMARIS reconstructions were carefully adjusted to fit original NRecon reconstructions. The total HEV length and thickness, branching points, and individual/average segment lengths were obtained by using the IMARIS Filament tracer module (Bitplane). Segments were defined as an HEV section between 2 branching points.

## RESULTS

### OPT CHARACTERIZATION OF HEV GROWTH AFTER IMMUNIZATION WITH OVA/CFA

**FIGURE 1:**
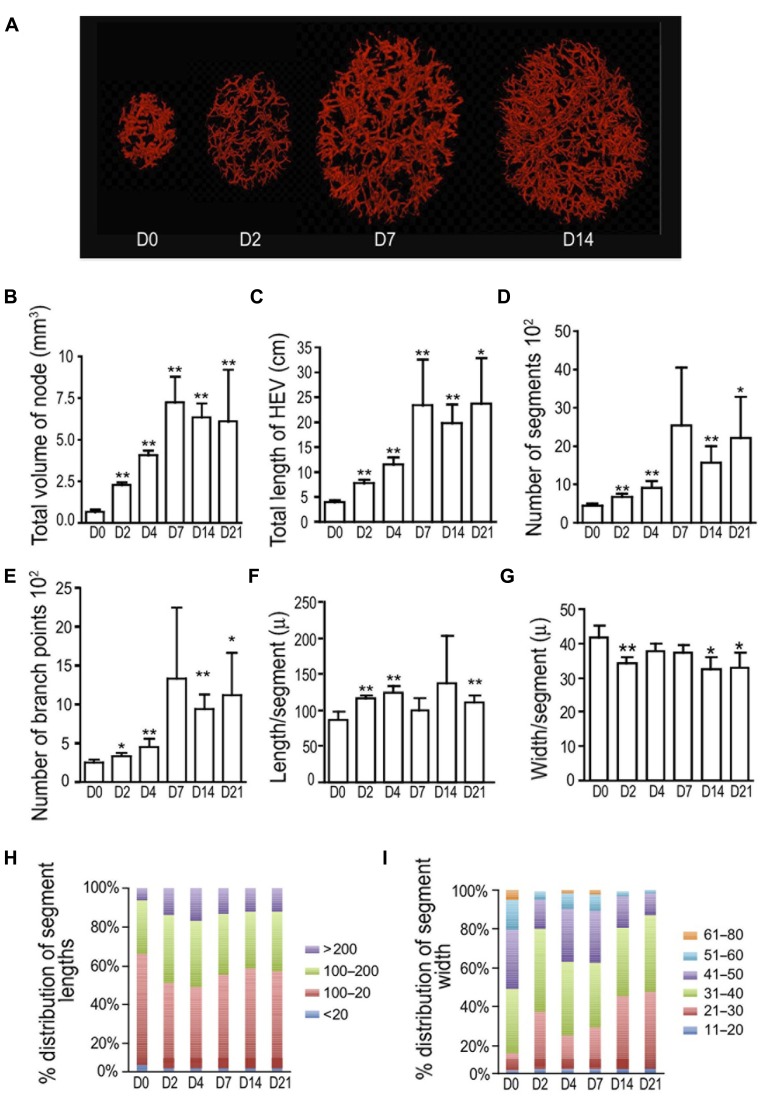
**Initial HEV lengthening is followed by branching and then narrowing after OVA/CFA.** Mice were immunized in the footpad with OVA/CFA on day 0 and draining popliteal nodes were prepared for OPT imaging of PNAd^+^ vessels on indicated days. **(A)** Representative images from OPT scanning. **(B)** Total lymph node volume. **(C)** Total length of HEV per lymph node. **(D)** Number of HEV segments per lymph node. **(E)** Number of branch points per lymph node. **(F)** Average segment length. **(G)** Average vessel width. **(H)** Relative distribution of segment lengths. Lengths were divided into discrete “bins” and the percent of segments in each bin was calculated and graphed. **(I)** Relative distribution of segment width. Widths were divided into discrete “bins” and the percent of segments in each bin was calculated and graphed. For **(B–G)**, *p < 0.05 and **p < 0.01 relative to day 0 measurement using *t*-test. Error bars represent standard deviations. *n* = 3–5 lymph nodes for each condition.

We used OPT to examine the morphologic alterations that occur as HEVs expand after immunization with OVA/CFA. The HEVs were labeled prior to imaging with intravenously injected fluorophore-tagged MECA-79, allowing us to visualize HEVs that express the PNAd epitope recognized by MECA-79 on the luminal surface. We examined the nodes at days 2, 4, 7, 14, and 21 to examine the morphologic alterations that occur during initiation, expansion, and the re-establishment of quiescence and stabilization. Popliteal lymph node volume was increased by day 2 after footpad immunization with OVA/CFA (**Figures 1A,B**), consistent with the rapid increase in lymph node cellularity observed previously (Webster et al., 2006; Tzeng et al., 2010; Chyou et al., 2011). Total HEV length doubled (**Figure 1C**). There was a modest increase in segment numbers as well as branch points (**Figures 1D,E**), suggesting that some of the HEV growth was attributable to the generation of new branches. The average segment length also increased, with the proportion of the longer segments increasing and segments over 200 μm doubling (**Figures 1F,H**). This suggested that HEV elongation contributed to HEV growth at day 2. HEV width was reduced at day 2 (**Figures 1G,I**), suggesting vasoconstriction or vessel stretching.

Lymph node volume and total HEV length increased further by day 4 (**Figures 1B,C**). Segment numbers and branch points showed continued increases while segment length did not increase from day 2 levels (**Figures 1D–F,H**). This suggested that the continued HEV growth at day 4 was mainly due to vessel arborization. Vessel width was restored to day 0 levels, further supporting the idea of HEV expansion (**Figures 1G,I**). At day 7, at a point when endothelial cell numbers are still expanding after OVA/CFA immunization (Tzeng et al., 2010), the combination of high segment numbers and branch points along with a reduction in segment length relative to that of day 4 (**Figures 1D–F,H**) suggested continued arborization and limited segment elongation. After day 7, when we have previously observed the re-establishment of vascular quiescence and stabilization (Tzeng et al., 2010), total HEV length, segment numbers, branch points, and segment length remained constant (**Figures 1C–F,H**) while vessel width decreased (**Figures 1G,I**), suggesting cessation of HEV growth and the process of vessel narrowing. Together, these results suggested that there is an initial HEV elongation and modest arborization in the first 2 days that is followed by increased HEV arborization and, after day 7, vessel narrowing.

### THE HEVs IN ACUTE AUTOINFLAMMATORY LYMPHADENOPATHY RESEMBLE HEVs AT DAYS 4–7 AFTER IMMUNIZATION WITH OVA/CFA

**FIGURE 2:**
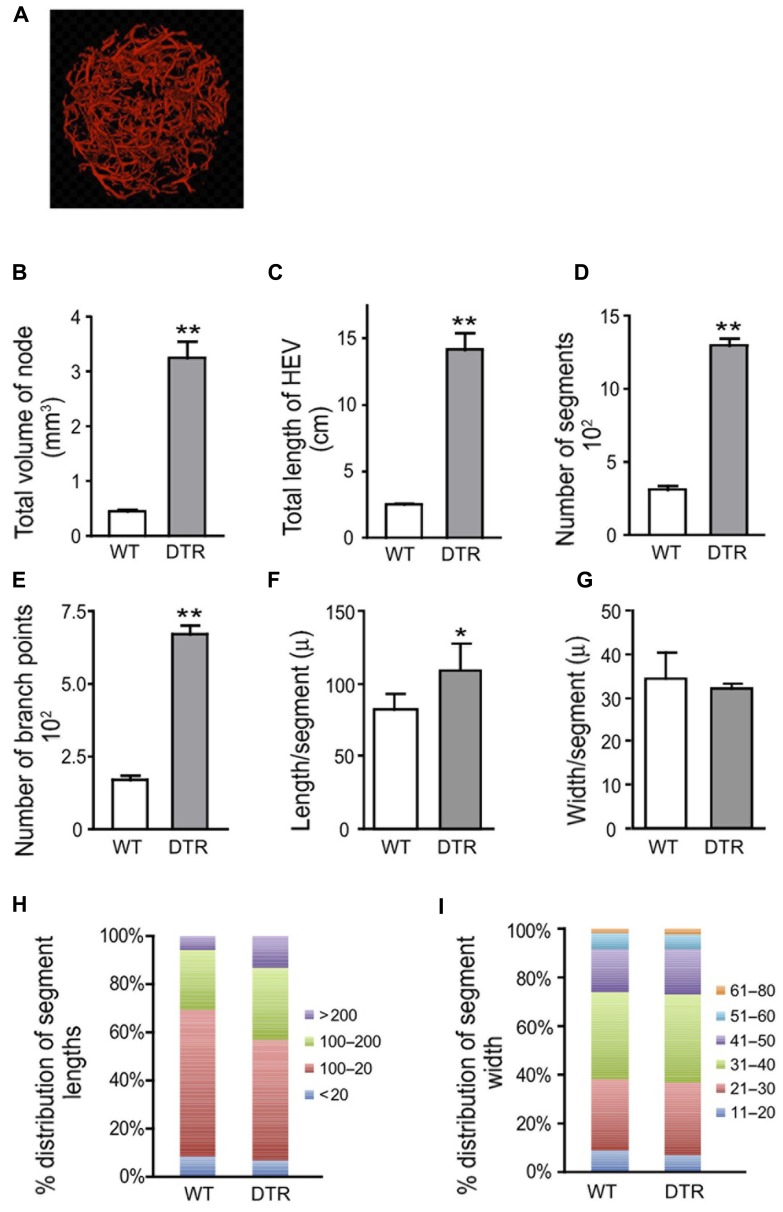
**High endothelial venule (HEV) growth in Foxp3-DTR mice is similar to that induced by OVA/CFA immunization.** Wild-type (WT) and Foxp3-DTR (DTR) mice were injected with DT to deplete regulatory T cells as per Section “Materials and Methods” and popliteal lymph nodes were prepared for OPT imaging of PNAd^+^ vessels. **(A)** Representative image of Foxp3-DTR lymph node from OPT scanning. Scale is same as that of **Figure 1A** to allow comparisons to nodes stimulated with OVA/CFA. **(B)** Total lymph node volume. **(C)** Total length of HEV per lymph node. **(D)** Number of HEV segments per lymph node. **(E)** Number of branch points per lymph node. **(F)** Average segment length. **(G)** Average vessel width. **(H)** Relative distribution of segment lengths. Lengths were divided into discrete “bins” and the percent of segments in each bin was calculated and graphed. **(I)** Relative distribution of segment width. Widths were divided into discrete “bins” and the percent of segments in each bin was calculated and graphed. For **(B–G)**, *p < 0.05 and **p < 0.01 relative to WT mice measurement using *t*-test. Error bars represent standard deviations. *n* = 4 lymph nodes for each condition.

We asked whether we could observe apparent vascular expansion in acutely enlarged lymph nodes associated with an autoimmune and autoinflammatory model and whether the expanded vasculature shared features with that induced by OVA/CFA immunization. Depletion of regulatory T cells in Foxp3-DTR mice by DT injection results in an acute autoimmune and inflammatory phenotype characterized by rampant inflammation, T cell activation, splenomegaly, lymphadenopathy, and autoantibody production by day 8 and death starting at day 10 (Kim et al., 2007; Chinen et al., 2010). As it is unknown whether the peripheral lymphadenopathy is directly driven by antigen-specific autoimmune responses or by the generalized autoinflammation, we will refer to the lymphadenopathy here as “autoinflammatory” in origin. We treated (control) wild-type mice and Foxp3-DTR mice with DT and examined lymph nodes at day 8. OPT analyses showed that lymph node volume, total HEV length, number of segments, number of branch points, and segment length were all increased in DT-treated Foxp3-DTR mice relative to wild-type mice (**Figures 2A–F,H**). Vessel width was similar in the WT and Foxp3-DTR mice (**Figures 2G,I**). The magnitude of the alterations in the Foxp3-DTR mice was similar to that at days 4–7 after OVA/CFA immunization (compare **Figures 1A–I** with **Figures 2A–I**). These results suggested that the HEVs in the acutely enlarged autoinflammatory lymph nodes were expanded and shared morphologic features with the expanded HEVs of lymph nodes acutely stimulated with OVA/CFA immunization.

**FIGURE 3:**
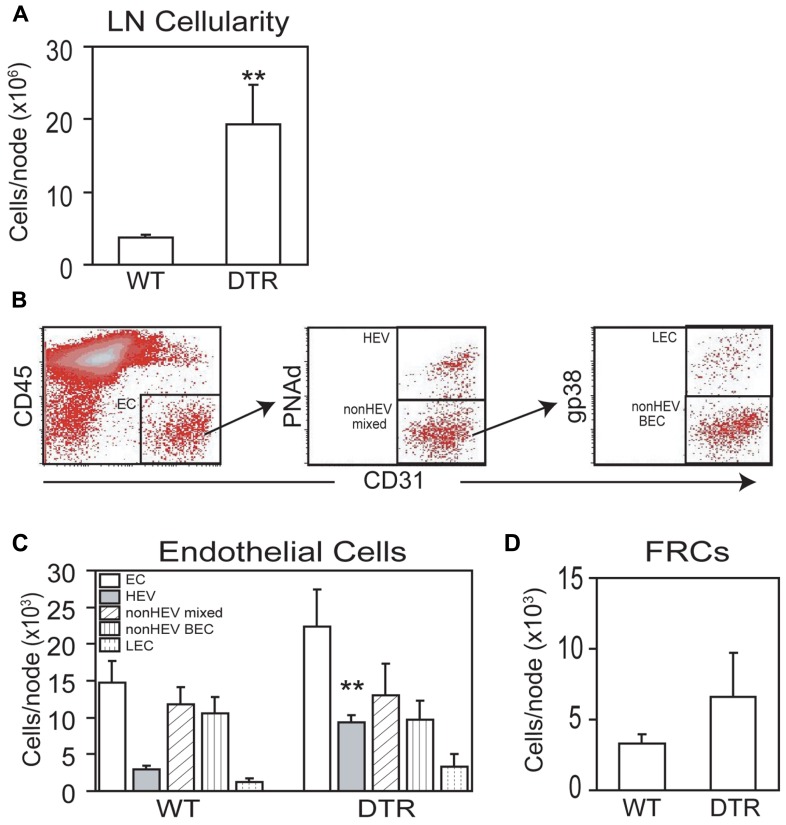
**Diphtheria toxin-treated Foxp3-DTR mice also shows more generalized lymph node vascular–stromal growth.** Wild-type (WT) and Foxp3-DTR (DTR) mice were injected with DT as per Section “Materials and Methods” and brachial lymph nodes were analyzed by flow cytometry. **(A)** Lymph node cellularity. **(B)** Flow cytometry plots to demonstrate gating of endothelial cell subpopulations. **(C)** Enumeration of endothelial cell subpopulations. **(D)** Enumeration of gp38^+^ FRCs per lymph node. For **(A,C,D)**, *p < 0.05, *t*-test, *n* = 3 mice per condition. For **(C)**, *t*-test is relative to the same endothelial cell subset in WT mice.

To further characterize vascular–stromal alterations in addition to HEV morphologic characteristics in the regulatory T cell-depleted mice, we enumerated endothelial cells and FRCs in the brachial nodes of the DT-treated Foxp3-DTR mice by flow cytometry. Lymph node cellularity was increased by four- to fivefold in the Foxp3-DTR brachial nodes (**Figure 3A**), a number fairly consistent with the six- to sevenfold increase in volume seen by OPT in the popliteal nodes (**Figure 2B**). As we have done previously, we gated as shown in **Figure 3B** to derive counts for endothelial cell subpopulations. CD45^-^CD31^+^ cells are “total endothelial cells” that can be subsetted into PNAd^+^ “HEV endothelial cells” and PNAd^-^ “non-HEV mixed” lymphatic and blood endothelial cells. The non-HEV mixed endothelial cells can be further subsetted into gp38^+^ “lymphatic endothelial cells” and gp38^-^ “non-HEV blood endothelial cells.” The three- to fourfold increase in HEV endothelial cell numbers in the Foxp3-DTR mice (**Figure 3C**) was consistent with the four- to fivefold increase in total HEV length observed in popliteal nodes using OPT (**Figure 2C**). In contrast to the HEV endothelial cells, the number of non-HEV blood endothelial cells was not increased in the Foxp3-DTR mice (*p* = 0.68, *t*-test, *n* = 3 per condition; **Figure 3C**). Lymphatic endothelial cells and (CD45^-^CD31^-^gp38^+^) FRCs showed a suggestion toward increased numbers in the Foxp3-DTR mice (**Figure 3C,D**), but the differences did not reach statistical significance (*p* = 0.12 for lymphatic endothelial cells; *p* = 0.14 for FRCs). These flow cytometry results corroborated the OPT results showing HEV expansion in the autoinflammatory lymph nodes and indicated that, at the time point examined, HEVs but not other subpopulations of the vascular–stromal compartment examined were expanded.

## DISCUSSION

In this study, we characterized the morphologic features of HEV expansion after OVA/CFA immunization and examined the HEVs in a model of acute autoinflammatory lymphadenopathy. We used OPT, which detects changes in HEVs expressing luminal PNAd. Because PNAd can be expressed both luminally and abluminally and it is possible for some HEVs to express only abluminal PNAd (Hemmerich et al., 2001; Drayton et al., 2003; Liao and Ruddle, 2006), OPT may miss remodeling involving cells expressing only abluminal PNAd. Nevertheless, the results reinforce the concept that there are distinct phases of lymph node vascular growth induced after immunization and demonstrate that acute autoinflammatory lymphadenopathy can be associated with HEV expansion. The HEV expansion with autoinflammation and morphologic similarities with HEVs in OVA/CFA-stimulated lymph nodes raise the possibility that insights gained from studying lymph nodes responding to exogenous stimuli may help to better understand vascular–stromal regulation in autoimmune and autoinflammatory diseases.

The initial elongation of HEV after OVA/CFA immunization followed by dominance of arborization corresponds with the findings of Anderson et al. (1975) who used intra-arterial Alcian blue dye and thick sections to study the lymph node vasculature. The initial segment elongation at day 2 could be due to either vessel stretching, as vessel width was reduced at this time point, or to the addition of PNAd^+^ endothelial cells to existing segments. In support of the latter, flow cytometric analysis showed a doubling of PNAd^+^ endothelial cell numbers at day 2 (Chyou et al., 2011). However, as flow cytometry is likely able to detect cells expressing only abluminal PNAd as well as cells expressing luminal PNAd, we cannot rule out that the additional PNAd^+^ cells detected by flow cytometry are cells expressing only abluminal PNAd and that elongation seen by OPT may simply reflect stretching. The subsequent increased arborization and expansion seen at days 4–7 corresponds well in time to the large increase in PNAd^+^ endothelial cell numbers that is seen during the expansion phase (Chyou et al., 2011). With OVA/CFA, endothelial cell numbers plateau at day 8 (Tzeng et al., 2010), similar to the plateau of HEV expansion seen with OPT at day 7. Given that the expansion of endothelial cell numbers is dependent primarily on B cells (Chyou et al., 2011), the dependence of branching expansion on B cells seen in the viral infection-induced model (Kumar et al., 2010) fits well with the idea that the increased arborization is the morphologic correlate of the increase in endothelial cell numbers seen during the lymphocyte-dependent vascular–stromal expansion phase. During the re-establishment of quiescence and stabilization after expansion, vessels become less leaky and FRCs reorganize more tightly around the vessels (Tzeng et al., 2010). The narrowing of vessels observed after day 7, then, may be the morphologic correlate of vascular stabilization. We have identified a role for CD11c^hi^ presumed dendritic cells in mediating the reduced permeability and reorganization of FRCs (Tzeng et al., 2010), and it will be of interest in future studies to understand whether the HEV narrowing is also dependent on CD11c^hi^ cells. Therefore, new data presented here add to our model of multiple phases of vascular expansion following peripheral immunization. Taking these data along with our previous studies, we propose that vascular expansion is comprised of (1) a CD11c^+^ cell-dependent, lymphocyte-independent initiation phase that is characterized by upregulation of vascular–stromal proliferation and a modest increase in HEV endothelial cell numbers resulting in HEV elongation and modestly increased arborization, (2) a lymphocyte-dependent phase characterized by continued vascular–stromal proliferation, generalized vascular–stromal expansion, and HEV growth that is mainly due to arborization, and (3) a CD11c^hi^ cell-dependent re-establishment of quiescence and stabilization that involves the re-accumulation of FRCs around vessels and consequent vessel narrowing.

Similar to the enlarged lymph nodes induced by immunization with OVA/CFA, enlarged lymph nodes in the DT-treated Foxp3-DTR mice also showed HEV expansion, lengthening, and branching. The magnitude of these changes at day 8 after the first injection of DT is similar to the changes at days 4–7 after OVA/CFA and this slight delay may be attributable to time required for regulatory T cell depletion. Consistent with the idea that these lymph nodes at day 8 are similar to a relatively early time point after exogenous immunization is the lack of non-HEV blood endothelial cell expansion, as HEV endothelial cells expand more rapidly than non-HEV blood endothelial cells after immunization with OVA/CFA or bone marrow-derived dendritic cells (Tzeng et al., 2010; Chyou et al., 2011). The death of animals by day 10 after regulatory T cell depletion (Kim et al., 2007) precludes satisfactory longer term analysis of the vascular–stromal growth to understand whether non-HEV endothelial cells and the rest of the vascular–stromal compartment also later expand, but it will be of interest to further examine the early events of HEV growth in the Foxp3-DTR mice to understand whether initial stages of HEV expansion is similar to that induced by OVA/CFA. Interestingly, HEV expansion at days 4–7 in the OVA/CFA model is similar to that at day 8 after LCMV infection in terms of total HEV length and segment numbers (Kumar et al., 2010), and the two models share a dependence on B cells (Kumar et al., 2010; Chyou et al., 2011) and multiple models share sensitivity to lymphotoxin beta receptor blockade (Liao and Ruddle, 2006; Kumar et al., 2010; Benahmed and Lu, unpublished observations) for induced HEV expansion. However, in the LCMV model, HEV branching precedes elongation (Kumar et al., 2010), suggesting stimulus-dependent differences in the initiation of HEV growth. The morphologic similarities of the expanded HEVs in the Foxp3-DTR mice with that after acute OVA/CFA immunization or viral infection (Kumar et al., 2010) suggests the possibility of at least some shared regulatory mechanisms.

We used regulatory T cell depletion as a means to induce a model of autoimmunity and autoinflammation, but it is possible that the associated lymph node vascular expansion reflects direct regulatory T cell activity on the vascular–stromal compartment. This would be consistent with studies that have implicated regulatory T cells in directly limiting endothelial cell activation (He et al., 2010; Matrougui et al., 2011). However, we have shown that CD11c^+^ cells are important for the initiation of lymph node vascular growth (Webster et al., 2006; Tzeng et al., 2010; Chyou et al., 2011). Regulatory T cell depletion results in the accumulation and activation of dendritic and other CD11c^+^ cells (Kim et al., 2007; Lund et al., 2008), raising the possibility that regulatory T cell depletion resulted in lymph node vascular expansion at least in part by promoting CD11c^+^ cell activation and accumulation.

Lymphadenopathy in diseases such as lupus and systemic onset juvenile arthritis can occur acutely with disease flare. Our results here suggest that acutely enlarged lymph nodes in the setting of an autoimmune and autoinflammatory model can have an expanded HEV compartment and that these HEVs share some similarities with those seen after immunization with exogenous stimuli. These results suggest the possibility that insights gleaned from vascular–stromal regulation in immunization models may be instructive for understanding how to therapeutically control immunity in autoimmune and autoinflammatory diseases.

## Conflict of Interest Statement

The authors declare that the research was conducted in the absence of any commercial or financial relationships that could be construed as a potential conflict of interest.
